# Counterfeit Artemisinin Derivatives and Africa: Update from Authors

**DOI:** 10.1371/journal.pmed.0040139

**Published:** 2007-03-27

**Authors:** Paul N Newton, Michael D Green, Facundo Fernandez

Since the publication of our article on counterfeit artesunate in June 2006 [1], further information has become available which we would like to report, as it has public health significance. An additional two counterfeit artesunate “types” with distinguishing features of the packaging have been found in mainland Southeast Asia, bringing the number of physical types to at least 14. For details see “Fake Artesunate Warning Sheet Number 5a” [2], an update (dated August 2006) to that published as supplementary material to the above paper.

In addition, we would like to bring readers' attention to the newspaper reports of counterfeit artesunate and dihydroartemisinin seized from ladies' handbags at Lagos airport [3].
